# Early Predictive Response to Multi-Tyrosine Kinase Inhibitors in Advanced Refractory Radioactive-Iodine Differentiated Thyroid Cancer: A New Challenge for [^18^F]FDG PET/CT

**DOI:** 10.3390/diagnostics11081417

**Published:** 2021-08-05

**Authors:** Cristina Ferrari, Giulia Santo, Rossella Ruta, Valentina Lavelli, Dino Rubini, Paolo Mammucci, Angela Sardaro, Giuseppe Rubini

**Affiliations:** 1 Nuclear Medicine Unit, Interdisciplinary Department of Medicine, University of Bari Aldo Moro, Piazza Giulio Cesare 11, 70124 Bari, Italy; ferrari_cristina@inwind.it (C.F.); giuliasanto92@gmail.com (G.S.); rossella.ruta@yahoo.it (R.R.); valentina.lavelli@gmail.com (V.L.); rubini.dino@libero.it (D.R.); paolo.mammucci@outlook.com (P.M.); giuseppe.rubini@uniba.it (G.R.); 2 Section of Radiology and Radiation Oncology, Interdisciplinary Department of Medicine, University of Bari Aldo Moro, Piazza Giulio Cesare 11, 70124 Bari, Italy

**Keywords:** RAI-R, differentiated thyroid cancer, multi-tyrosine kinase inhibitors, positron emission tomography, PET, [^18^F]FDG PET/CT

## Abstract

Differentiated thyroid cancer (DTC) represents the most common thyroid cancer histotype. Generally, it exhibits a good prognosis after conventional treatments; nevertheless, about 20% of patients can develop a local recurrence and/or distant metastasis. In one-third of advanced DTC, the metastatic lesions lose the ability to take up iodine and become radioactive iodine-refractory (RAI-R) DTC. In this set of patients, the possibility to perform localized treatments should always be taken into consideration before the initiation of systemic therapy. In the last decade, some multi-tyrosine kinase inhibitor (MKI) drugs were approved for advanced DTC, impacting on patient’s survival rate, but at the same time, these therapies have been associated with several adverse events. In this clinical context, the role of 2-deoxy-2-[^18^F]fluoro-D-glucose positron emission tomography/computed tomography ([^18^F]FDG PET/CT) in the early treatment response to these innovative therapies was investigated, in order to assess the potentiality of this diagnostic tool in the early recognition of non-responders, avoiding unnecessary therapy. Herein, we aimed to present a critical overview about the reliability of [^18^F]FDG PET/CT in the early predictive response to MKIs in advanced differentiated thyroid cancer.

## 1. Introduction

Thyroid cancer (TC) ranked 9th place for incidence among cancers in 2020, being responsible for 3.0% of all cancers and 0.4% of cancer-related deaths [1]. Its incidence is threefold higher in women than in men, with rates varying widely from country to country [2]. The etiology of TC is not clearly defined, with the only established risk factor represented by ionizing radiation, in particular when exposure happens in childhood [1]. In the last three decades, a rapid and steady worldwide increase in TC’s incidence has been observed, with a stable or slight decline in mortality rates. The observed trend has been mainly attributed to the progressive improvements in diagnostic modalities, including imaging techniques and biopsy procedures, that facilitate cancer detection even in subclinical and/or indolent states. Nevertheless, the subsequent overdiagnoses have been accompanied by a relatively stable mortality rate, as not all diagnosed cancers require treatments and active surveillance is enough in the majority of cases [2].

Management and treatment options differ on the basis of the TC type and its clinical stage. The most common histotype is the differentiated thyroid cancer (DTC), comprehending the papillary thyroid cancer (PTC), making up about 85% of all DTCs; the follicular thyroid cancer (FTC), 12%; and a smaller percentage (3%) of Hurtle cell cancer (HTC). DTC generally exhibits a good prognosis after conventional treatments, including surgical resection, radioactive iodine (RAI) administration, and TSH suppression therapy; nevertheless, about 20% of patients can develop a local recurrence (thyroid bed or cervical lymph nodes) and distant metastasis is detected in 10% of cases. In this set of patients, multi-tyrosine kinase inhibitors (MKIs), new drug therapies with a molecular target, have been proposed, showing significant levels of disease control in advanced refractory DTC patients [3,4]. Namely, phase III trials showed that these drugs impact progression-free survival (PFS) significantly (10.8 vs. 5.8 months for sorafenib; 18.3 vs. 3.6 for lenvatinib). However, these innovative therapies have been associated with several adverse events (AEs), thus an overall survival (OS) benefit has not been demonstrated yet. It follows that precise and early recognition of non-responders is essential, avoiding unnecessary therapy. In this clinical scenario, 2-deoxy-2-[^18^F]fluoro-D-glucose positron emission tomography/computed tomography ([^18^F]FDG PET/CT) is considered a sensitive and specific diagnostic method that could help the evaluation of TC’s response to these innovative therapies used in advanced stages of the disease. 

The aim of this review is to evaluate the reliability of [^18^F]FDG PET/CT in the early predictive response to MKIs in advanced differentiated thyroid cancer.

## 2. Advanced Refractory DTC and MKIs Employment Rationale

In one-third of advanced DTC, the metastatic lesions lose the ability to take up iodine (RAI-refractory DTC, RAI-R DTC) with no more RAI efficacy [5] and a significant impact on the 10 year OS rate which falls to 60%, and <50% for stage III and IV, respectively [6]. To date, the possibility to perform localized treatments in patients with metastatic DTC, that progresses despite standard therapies, should always be taken into consideration before the initiation of systemic treatment [7]. Adjuvant neck locoregional external beam radiotherapy (EBRT) should be considered for those patients who undergo neck re-operations for palliation of locoregional recurrences and in some cases of bone metastases for pain control. Recently, other localized treatments with thermal (radiofrequency or cryo-) ablation, ethanol ablation, or chemo-embolization have been evaluated on single/few metastases or on locoregional persistence/recurrence of neoplastic disease. Systemic cytotoxic chemotherapy is not routinely used with Doxorubicin as the only drug approved [8].

Nowadays, targeted therapies inhibiting abnormally activated tyrosine kinase (TK), represent the systemic therapies that should be used as first-line treatment in metastatic DTCs. The rationale for the development of new “ad hoc” therapies for this subgroup of patients derives mainly from a set of mutations that concern two predominant signaling pathways: mitogen-activated protein kinase (MAPK) and phosphatidylinositol-3 kinase (PI3K) pathways, including mutations in central mediators such as BRAF, RAS, PTEN, AKT, and PI3KCA [9,10,11]. When a mutation occurs as a result of a somatic or germline genetic alteration of the transmembrane receptors (TKR) or from a central mediator, the signaling becomes constitutively activated. The phosphorylation of many intracellular proteins involved in the signal transduction cascade enhances the catalytic activity, responsible for uncontrolled cell growth, loss of cell differentiation, and a decrease of cell apoptosis [8].

BRAFV600E is the most common mutation described in 18–87% of PTCs [12,13]. Interestingly, it is mutually exclusive with other mutations, suggesting that the presence of one single driver mutation is sufficient for thyroid tumorigenesis [14]. BRAFV600E mutation, as well as the oncogenic activation of MEK/ERK, also plays an important role in the loss of sodium iodide symporter (NIS) activity, leading to RAI refractoriness [15]. RAS mutations are reported in 30–40% of FTC, in 30–45% of follicular variant PTC (rarely in the classical variant of PTC), and in 15% of HTC [16,17,18,19]. PTEN, AKT, and PIK3CA are the most commonly mutated mediators in PI3K pathways, although their frequency is low [20]. RET proto-oncogene is one of the most commonly altered oncogene codings for a TKR that, when mutated, becomes constitutively activated with subsequent activation of MAPK and PI3K pathways [16]. 

### 2.1. Multi-Kinase Inhibitors in Advanced RAI-R DTCs

Over the last decade, several molecular agents able to inhibit TK and TKR with different mechanisms have been generated [21]. TKIs are able to inhibit specific oncogene alterations but they also act against several other TKRs and for this reason they are also recognized as MKIs. As is known, the expression of both thyroid-specific genes and thyroid-specific transcription factors decreased in de-differentiated thyroid cancers [22]. Interestingly, MKIs, thanks to their ability to target different molecules, could be able to restore radioiodine avidity, re-differentiating NIS expression by activating different pathways [15,23]

Among MKIs, sorafenib and lenvatinib have finally been approved by both the Food and Drug Administration (FDA) and the European Medical Agency (EMA) for the treatment of advanced RAI-R DTC. Sorafenib has been the first FDA-approved MKI, which targets VEGFR 1, 2, and 3, PDGFR, Raf-1, RET, and BRAF. The drug’s efficacy has been demonstrated by the phase III DECISION study, paving the way for this new target therapy in DTC [24]. The second one lenvatinib, is an oral drug targeting VEGFRs 1–3, FGFRs 1–4, PDGFR α, RET, and c-KIT signaling networks [25], approved in 2015 following the placebo-controlled phase III SELECT study results [26].

Several other MKIs have been tested for the treatment of advanced, progressive DTC patients who no longer benefit from RAI, with a PR rate varying from 8% to 64.7% [24,26,27,28], but they have not been approved yet. Apatinib, whose efficacy and safety has already been proved in gastric cancer [29], is a small-molecule MKI that inhibits VEGFR-2, leading to the inhibition of VEGF-mediated endothelial cell migration and proliferation and a decrease in tumor microvascular density [30]. Sunitinib is currently approved for the therapy of renal cell carcinoma and gastrointestinal stromal tumor [31]. It is an MKI whose targets include VEGFRs 1 and 2, PDGFR, c-KIT, FLT3, and RET. Point mutations and rearrangements of RET are present in 2–60% of patients with PTC, making sunitinib a possible rational candidate for therapy [28]. 

Sometimes, the MKI’s activity against these other receptors is stronger than the activity they have against the driver oncogene alteration of TC, and several AEs can occur because of these “off-targeted” activities. 

### 2.2. MKIs Related-Adverse Events

MKIs such as sorafenib and lenvatinib, among others, have been demonstrated to have an impact on disease progression, but are limited by a wide range of toxicities. For these reasons, patients must be strictly monitored especially during the first 6 months of therapy. Hypertension is a frequent side effect and patients should check their blood pressure frequently in order to allow closer monitoring, eventually followed by a personalized antihypertensive therapy. Diarrhea affects around 80% of patients and is likely one of the most disabling AEs when the degree of severity is high. Cutaneous alterations, such as the palmar-plantar erythrodysaesthesia syndrome (“hand-foot syndrome”), are extremely frequent under sorafenib, being the most common reason for dose reduction, interruption, and withdrawal. Weight loss, fatigue, anorexia, nausea, proteinuria, alopecia, and increased TSH values are other frequent AEs in patients treated with sorafenib and lenvatinib according to phase II, phase III registration, and real-life studies [24,32,33,34,35].

The most recent American Thyroid Association (ATA) recommendations state the MKIs indications in RAI-R DTC patients with metastatic, rapidly progressive, symptomatic, and/or imminently threatening disease not otherwise amenable to local control using other approaches [36,37,38], but there is no common agreement on the right time to start MKI therapy. A discontinuation rate of 20% related to AEs has been estimated. In the pioneer phase III SELECT trial, the discontinuation rate of lenvatinib because of AEs during the treatment course was 14.2% [26]. Similar results have been obtained with sorafenib in the phase III DECISION trial. Notably, drug toxicity has led to dose modification in 78% of patients and permanent discontinuation of therapy in 19% [24]. 

For these reasons, the optimal time to start MKIs in patients with RAI-R DTC remains controversial. Some authors studied several parameters, including tumor burden, disease progression, symptoms, and a high risk of local complications for selecting patients suitable to systemic treatment [39]. Tumor burden was evaluated by Suzuki et al. who demonstrated a better survival outcome when lenvatinib was started with a baseline sum tumor diameter of the target lesion (SumTLs) < 60 mm or a maximum tumor diameter of the target lesion (MaxTL) < 34 mm [40].

Based on these data and with increasing clinical experience, a judicious MKI initiation is warranted and the early prediction of tumor response and prognosis is of great importance for the optimization of the patient’s treatment. 

## 3. [^18^F]FDG PET/CT in Early MKIs Prediction of Response

Functional imaging is an essential diagnostic tool because of its ability to define biological tumor characteristics and provide useful information for prognostic and therapeutic purposes. In the thyroid, follicular epithelial cells exhibit avid iodine uptake mediated by the NIS. The partially conserved activity of this transporter in aberrant cells of DTC is the basis for the successful therapeutic and diagnostic utility of RAI [41]. Poorly differentiated carcinomas do not express the same molecular features, requiring the employment of alternative radiotracers. The use of [^18^F]FDG has been shown to be a troubleshooter thanks to the increased glucose uptake through overexpression of the glucose transporter-1 (GLUT 1) in thyroid cells during dedifferentiation. The inverse relationship between iodine uptake and glucose utilization, called the iodine/FDG “flip-flop phenomenon”, reflects the cell differentiation status and heterogeneous pattern of NIS expression [42,43]. The ATA recommendations for the use of [^18^F]FDG PET/CT are almost exclusively for patients with a high risk of metastasis or recurrence presenting with increasing thyroglobulin levels (>10 ng/mL) and a negative RAI scan [36]. 

Nowadays, with the introduction of new therapies for metastatic patients, [^18^F]FDG PET/CT is being tested to become a reference imaging tool in selected patients. In particular, scientific evidence in recent years is focusing on its role in predicting response to new MKI therapies. The chance to predict the response just a few days after starting therapy represents a promising challenge for PET imaging. In this context, accurate assessment of treatment response is crucial to discriminate between responders and non-responders, avoiding inefficient therapy and its potential AEs. 

The current standard for monitoring tumor response is the measurement of change in tumor size on morphological imaging techniques such as CT, based on the Response Evaluation Criteria in Solid Tumors (RECIST)[44], which have been updated (RECIST 1.1 criteria) in 2009 [45]. As newer cancer treatments are cytostatic rather than cytotoxic, functional changes are expected to precede the morphologic ones and therefore [^18^F]FDG PET/CT has the potential to improve the diagnostic accuracy and prediction of tumor course [46] Moreover, [^18^F]FDG reflects glucose metabolism, which is controlled by the PI3K/AKT pathways, also known as the protein kinase B. As lenvatinib inhibits this pathway upstream, its therapeutic effects can be detected as [^18^F]FDG uptake decreases on PET/CT [47,48]. In 2009, Wahl et al. described the Positron Emission Tomography Response Criteria In Solid Tumors (PERCIST) to provide structured guidance for response assessment using [^18^F]FDG PET/CT, subsequently modified in a PERCIST 1.0 version [48]. However, no specific recommendations for the use of PERCIST in DTC response assessment outside clinical trials have been reported. Few studies assessing MKIs response by [^18^F]FDG PET/CT are available, but the emerging results are promising. The most important studies evaluated are reported in Table 1.

In 2010, Carr LL et al. conducted an exploratory analysis of early [^18^F]FDG PET/CT and tumor marker responses on 24 patients. The metabolic parameters were correlated with radiographic response and time-to-tumor progression (TTP). [^18^F]FDG PET/CT was performed both at baseline and after seven days of “off-label” Sunitinib therapy. At baseline, the median average Standardized Uptake Value (SUV) and SUV for the most avid lesion were 7.9 (SD = ±16.2; range 3.3 to 59.6) and 13.0 (SD = ±20.5; range 3.8 to 67.0), respectively. The median percent average SUV change from baseline to the first time-point evaluation was −11.7% in patients who had a RECIST response, and −13.9% in patients with stable disease (SD), versus 8.6% in those with progressive disease (PD), confirming that [^18^F]FDG PET/CT was an early indicator of response to sunitinib just one week after starting MKI therapy [28].

**Figure 1:**
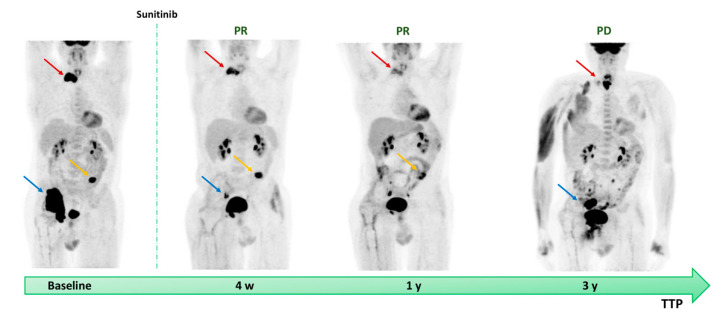
A 52 year-old man affected by metastatic RAI-R DTC. The patient underwent [^18^F]FDG PET/CT before starting “off-label” Sunitinib therapy. The baseline PET showed high uptake in the transverse process of D1 (red arrow), left iliac wing (yellow arrow), and in the right hip, involved adjacent soft tissue (blue arrow). The patient first underwent right hip resection and reconstruction with a graft, then started MKI therapy and repeated PET exam after 28 days from the starting of Sunitinib with partial response. No AEs were reported and the patient continued target therapy. The one-year follow-up showed a significative response to Sunitinib therapy and a time to progression of three years. PR = partial response; PD = progression disease; w = week; y = year; TTP = time to tumor progression; RAI-R = refractory radioactive iodine; [^18^F]FDG PET/CT = 2-deoxy-2-[^18^F]fluoro-D-glucose positron emission tomography/computed tomography; MKIs = multi-kinase inhibitors; AEs = adverse events.

The clinical case reported in Figure 1 shows the [^18^F]FDG PET/CT predictive response to Sunitinib therapy.

The role of serum thyroglobulin (Tg) and [^18^F]FDG PET/CT was evaluated in another study using sorafenib therapy [49]. The authors reported that baseline Tg levels were significantly higher in patients who showed PD compared with responders. Furthermore, there was a strong correlation between both baseline and post-therapy Tg with PFS. In addition, regarding [^18^F]FDG PET/CT assessment, the baseline average SUVmax was significantly higher in patients who showed PD (SUVmax > 3 in all cases) compared with responding subjects, but no significant correlation with PFS was found. At the early [^18^F]FDG PET/CT evaluation, the average SUVmax reductions were more robust in patients who achieved clinical benefit from treatment compared with non-responding subjects [49].

Ahmaddy et al. investigated the role of [^18^F]FDG PET/CT for monitoring functional tumor response in comparison to morphological imaging and its ability to predict PFS and disease-specific survival (DSS) in patients with advanced RAI-R DTC undergoing lenvatinib treatment. In this cohort, patients with PD at the 3-month follow-up evaluated by mPERCIST, showed a significantly lower median PFS (4.0 vs. 24 months, *p* = 0.008) and DSS (20.0 months vs. median not reached for responders, *p* = 0.015) compared to disease control (DC). Similar results were achieved by mPERCIST evaluation at the 6-month follow-up (PFS 4.0 vs. 15.0 months, *p* = 0.003). Moreover, all responders showed a decline in all PET-semiquantitative parameters, whereas a lack of functional tumor response was associated with a worse outcome. SUVpeak, SUVmax, metabolic tumor volume (MTV), and total lesion glycolysis (TLG) at the 3 and 6-month follow-ups were significantly higher in patients with PD and could serve as additional markers for monitoring early tumor response and outcome [50]. 

On the other hand, according to RECIST criteria, patients with PD showed a significantly lower median DSS at both time-points, without any significant correlation with PFS, demonstrating that functional imaging is able to capture early treatment effects that could be underestimated by morphological imaging only. Notably, as for other solid tumors (i.e., renal cell carcinoma), reduction in lesion size was not shown to be an accurate predictor of a good response to MKIs by itself since responding lesions sometimes showed an increase in size, despite evidence of low FDG uptake and high PFS [51,52]. 

**Figure 2:**
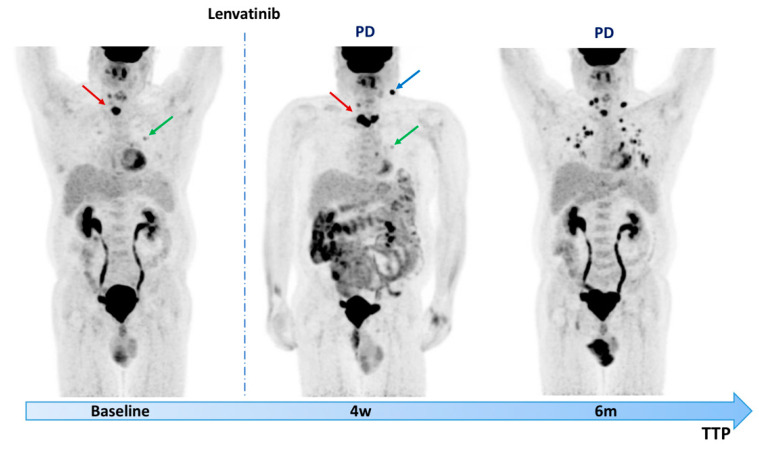
A 63-year-old man with metastatic RAI-R well-differentiated follicular cell thyroid cancer underwent [^18^F]FDG PET/CT before starting lenvatinib therapy. Baseline PET showed an intense FDG uptake in the paratracheal region, corresponding to locoregional disease and moderate uptake in the bilateral pulmonary field. The patient then underwent reevaluation after 28 days from the beginning of MKI therapy, which showed disease progression with the appearance of a metastatic submandibular lymph node. After surgical lymph node resection, the patient continued lenvatinib therapy with disease progression 6 months later. PD = progression disease; w = week; m = month; TTP = time to tumor progression; RAI-R = refractory radioactive iodine; [^18^F]FDG PET/CT = 2-deoxy-2-[^18^F]fluoro-D-glucose positron emission tomography/computed tomography; MKIs = multi-kinase inhibitors.

Figure 2 shows [^18^F]FDG PET/CT predictive evaluation at 4 weeks indicating disease progression confirmed at the 6-month follow-up. 

Eight weeks following apatinib administration, [^18^F]FDG PET/CT parameters’ correlation with RECIST 1.1 and Tg level were evaluated in another study conducted by Lin et al. Nine out of ten patients achieved PR and one patient achieved SD, according to the RECIST 1.1 criteria. Taking into account the small sample size, the disease control rate (DCR) and objective response rate (ORR) of the study were 100% (10/10) and 90% (9/10), respectively. A significant decrease in SUVmax was revealed after 8 weeks of treatment, indicating a rapid decrease in glucose metabolism in target lesions. Interestingly, the SUVmax diminished from 6.53 ± 5.14 to 2.56 ± 1.67 (*p* = 0.032) and 2.45 ± 1.48 (*p* = 0.020) after 4 and 8 weeks of treatment, respectively. Whilst no correlation was found between changes in Tg level and SUVmax (after 8 weeks) or Tg level and total tumor diameter (TTD), a linear correlation was demonstrated for change in diameter at 8 weeks post-therapy and SUVmax at both 4 and 8 weeks evaluation in the target lesions (*p* = 0.009 and 0.036, respectively). As a result, Tg exhibited the most important and fastest decrease, followed by glucose metabolism revealed by PET/CT and TTD reflected by CT. Furthermore, a more significant decrease in SUVmax was observed than structural change reflected by RECIST (60.8% vs. 34.0%), implying an earlier and more pronounced metabolic response to apatinib [30].

In a later study, the same authors evaluated [^18^F]FDG PET/CT response to apatinib in a larger population by using the European Organization for Research and Treatment of Cancer (EORTC) PET study group criteria too [53]. They performed a PET scan at 28 days after one cycle of MKI therapy. A strong correlation was found between the changes in the sum of the longest diameters of target lesions (ΔCT%) and ΔMTV%, ΔTLG% and ΔSUVmax% both in one-cycle and two-cycle treatments. The authors suggested that this strong correlation could lead to the use of ΔMTV% or ΔTLG% at the first cycle to predict the shrinkage of tumor lesions [53]. 

In a subset of patients who received MKI therapy, Manohar et al. demonstrated that the log-MTV values tended to be higher compared to other treatment strategies. Moreover, MKI therapy was associated with significantly higher OS rates [54]. 

Even if encouraging, the routinary use of volume-based PET parameters in MKI therapy responses in advanced DTC patients needs to be further investigated in a larger study population, before replacing the consolidated role of SUVmax in the clinical practice. 

Interestingly, as it emerges from a recent Italian study, a longer OS in patients with a metabolic response at [^18^F]FDG PET/CT performed after 4 weeks of lenvatinib treatment was reached (36.53 vs. 11.28 months) [55]. On these premises, it is speculated that [^18^F]FDG PET/CT could also serve as an imaging biomarker for the prognosis of OS and PFS.

## 4. Future Challenges

As for other solid cancers, increasing evidence suggests the potential role of immune-checkpoints, such as cytotoxic T-cell antigen4 (CTLA-4), programmed cell death protein 1 (PD-1), and programmed cell death protein ligand 1 (PD-L1) in TC. Different PD-L1 expression was demonstrated, depending on the histology, with even major expression in advanced TC [56]. In PTC, greater expression of CTLA-4 and PD-L1 is associated with BRAFV600E mutation and a lower degree of differentiation [57]. Even in this context, [^18^F]FDG PET/CT could represent an essential diagnostic tool for treatment response evaluation [8].

Literature data about MKI therapy evaluation with other radiopharmaceuticals are anecdotal but interesting. Basu et al. reported the case of a 57-year-old man with metastatic localizations of RAI-R DTC in the left fibula under sorafenib treatment. The metastasis was positive in [^68^Ga]DOTATATE PET/CT and showed a high uptake in [99mTc]Tc-HYNIC-TOC scintigraphy. Consequently, supposing that thyroid cancer expressed somatostatin receptors, authors used [^68^Ga]DOTATATE PET/CT for treatment response evaluation [58] 

[^68^Ga]-labelled RGD PET/CT imaging for pre- and post-treatment evaluation was also evaluated for its ability to mimic anti-angiogenesis drugs. In particular, [^68^Ga]-NOTA-PRGD2 was found to be not only effective in the evaluation of response, but also a potential predictor of prognosis after MKI therapy [53]. 

## 5. Conclusions

The introduction of MKIs in metastatic RAI-R DTC is leading to a paradigm shift in the treatment of the disease, providing new opportunities when other therapies fail. However, correlated AEs often impact patient management and therapy success. [^18^F]FDG PET/CT, with the added value of semiquantitative analysis, could offer in-vivo biochemical evidence for early assessment of MKIs, helping decision-making to avoid unnecessary therapies. However, the specific timing of “early evaluation” is still under debate with the 4–8 week treatment response evaluation representing the most fascinating one, for which it is worth investigating further. Prospective studies and further research are needed to elicit if [^18^F]FDG PET/CT can detect phenotypically aggressive tumor types, identifying patients that would benefit from early initiation of MKI therapy.

## Figures and Tables

**Table 1:** Characteristics of the original articles reviewed.

Author-PMID	Journal- Year	MKIs	PET Timing	PET Parameters	Main Findings
Ahmaddy et al. 33467085	Cancers 2021	lenvatinib	3 v 6 mo	mPERCIST; SUVmax; SUVmean; MTV; TLG; SUVpeak	All RECIST responders showed a decline in PET-parameters. Non-responders according to mPERCIST showed significantly lower median PFS and DSS, whereas according to RECIST, only DSS was significantly lower.
Wang et al. 29618578	Endocrine-Rel Canc. 2018	apatinib	baseline; 4 weeks; 8 weeks	EORTC; ΔMTV; ΔTLG	A strong correlation between the best change in ΔCT% and [^18^F]FDG PET/CT parameters after the first treatment cycle (ΔMTV%, ΔTLG%, and ΔSUVmax%). A significant difference in PFS was observed between patients with ΔMTV% < −45% and ≥−45% and between patients with ΔTLG% < −80% and ≥−80%.
Lin et al. 28178685	Oncotarget 2017	apatinib	baseline; 4 weeks 8 weeks	SUVmax	A significant decrease in SUVmax after 8 weeks of treatment was found. Linear correlations of change in diameter after 8 w of therapy with changes in SUVmax after 4/8 w of therapy were observed in the target lesions.
Carr et al. 20847059	Clin Cancer Res. 2010	sunitinib	baseline; 7 days	SUVmax	A statistically significant association of TTP with increases in average % SUV was found. The risk of tumor progression increased by 4% for each 1% increase in average SUV from baseline.
Marotta et al. 23009688	Clin Endocrinol 2013	sorafenib	baseline; 15 days	SUVmax	Baseline average SUVmax was significantly higher in patients who showed DP, but no significant correlation with PFS was found. Early [^18^F]FDG-PET scans showed a reduction in average SUVmax in all responders.
Manohar et al. 30015659	Clin Nucl Med. 2018	N.D.	baseline	log-MTV; log-TLG	The log-MTV values for patients on MKIs therapy tended to be higher than with other therapies. MKIs therapy was associated with significantly higher OS rates.

MKIs = Multi-kinase inhibitors; mPERCIST = Modified Positron Emission Tomography Response Criteria In Solid Tumors; SUV = standardized uptake value; MTV = metabolic tumor volume; TLG = total lesion glycolysis; RECIST = Response Evaluation Criteria in Solid Tumors; PFS = progression free survival; DSS = disease-specific survival; EORTC = European Organization for Research and Treatment of Cancer EORTC; ΔCT = longest diameters of target lesions; [^18^F]FDG PET/CT = 2-deoxy-2-[^18^F]fluoro-D-glucose positron emission tomography/computed tomography; Tg = thyroglobulin; TTP = time to tumor progression; DP = disease progression; OS = overall survival; N.D. = not define.

## Abbreviations

PI3K	phosphatidylinositol-3 kinase
MAPK	mitogen activated protein kinase
RAS	rat sarcoma
PTEN	phosphatase and tensin homolog
AKT	protein kinase B
RAF	rapidly accelerated fibrosarcoma
FGFR	fibroblast growth factor receptors
FLT3	Fms Related Receptor Tyrosine Kinase 3
EGFR	Epidermal Growth Factor Receptor
TKR	tyrosine kinase receptor
VEGFR	vascular endothelial growth factor receptor
PDGFR	platelet derived growth factor receptor
RET	rearranged during transfection
TSH	thyroid stimulating hormone

## References

[1] Sung H., Ferlay J., Siegel R.L., Laversanne M., Soerjomataram I., Jemal A., Bray F. (2021). Global cancer statistics 2020: GLOBOCAN estimates of incidence and mortality worldwide for 36 cancers in 185 countries. CA A Cancer J. Clin..

[2] Filetti S., Durante C., Hartl D., Leboulleux S., Locati L.D., Newbold K., Papotti M.G., Berruti A. (2019). Thyroid cancer: ESMO clinical practice guidelines for diagnosis, treatment and follow-up. Ann. Oncol..

[3] Schmidt A., Iglesias L., Klain M., Pitoia F., Schlumberger M.J. (2017). Radioactive iodine-refractory differentiated thyroid cancer: An uncommon but challenging situation. Arch. Endocrinol. Metab..

[4] Jayarangaiah A., Sidhu G., Brown J., Campbell O., McFarlane S. (2019). Therapeutic options for advanced thyroid cancer. Int. J. Clin. Endocrinol. Metab..

[5] Durante C., Haddy N., Baudin E., Leboulleux S., Hartl D., Travagli J.P., Caillou B., Ricard M., Lumbroso J.D., de Vathaire F. (2006). Long-term outcome of 444 patients with distant metastases from papillary and follicular thyroid carcinoma: Benefits and limits of radioiodine therapy. J. Clin. Endocrinol. Metab..

[6] Nam S.H., Bae M.R., Roh J.L., Gong G., Cho K.J., Choi S.H., Nam S.Y., Kim S.Y. (2018). A comparison of the 7th and 8th editions of the AJCC staging system in terms of predicting recurrence and survival in patients with papillary thyroid carcinoma. Oral Oncol..

[7] Haugen B.R. (2017). 2015 American thyroid association management guidelines for adult patients with thyroid nodules and differentiated thyroid cancer: What is new and what has changed?. Cancer.

[8] Lorusso L., Cappagli V., Valerio L., Giani C., Viola D., Puleo L., Gambale C., Minaldi E., Campopiano M.C., Matrone A. (2021). Thyroid cancers: From surgery to current and future systemic therapies through their molecular identities. Int. J. Mol. Sci..

[9] Prete A., Borges de Souza P., Censi S., Muzza M., Nucci N., Sponziello M. (2020). Update on fundamental mechanisms of thyroid cancer. Front. Endocrinol..

[10] Agrawal N., Akbani R., Aksoy B.A., Ally A., Arachchi H., Asa S.L., Auman J.T., Balasundaram M., Balu S., Baylin S.B. (2014). Integrated genomic characterization of papillary thyroid carcinoma. Cell.

[11] Elisei R., Ugolini C., Viola D., Lupi C., Biagini A., Giannini R., Romei C., Miccoli P., Pinchera A., Basolo F. (2008). BRAFV600E Mutation and outcome of patients with papillary thyroid carcinoma: A 15-year median follow-up study. J. Clin. Endocrinol. Metab..

[12] Ganly I., Makarov V., Deraje S., Dong Y.Y., Reznik E., Seshan V., Nanjangud G., Eng S., Bose P., Kuo F. (2018). Integrated genomic analysis of hürthle cell cancer reveals oncogenic drivers, recurrent mitochondrial mutations, and unique chromosomal landscapes. Cancer Cell.

[13] Kebebew E., Weng J., Bauer J., Ranvier G., Clark O.H., Duh Q.Y., Shibru D., Bastian B., Griffin A. (2007). The prevalence and prognostic value of braf mutation in thyroid cancer. Ann. Surg..

[14] Ciampi R., Romei C., Pieruzzi L., Tacito A., Molinaro E., Agate L., Bottici V., Casella F., Ugolini C., Materazzi G. (2017). Classical point mutations of ret, braf and ras oncogenes are not shared in papillary and medullary thyroid cancer occurring simultaneously in the same gland. J. Endocrinol. Investig..

[15] Chakravarty D., Santos E., Ryder M., Knauf J.A., Liao X.H., West B.L., Bollag G., Kolesnick R., Thin T.H., Rosen N. (2011). Small-molecule mapk inhibitors restore radioiodine incorporation in mouse thyroid cancers with conditional braf activation. J. Clin. Investig..

[16] Tirrò E., Martorana F., Romano C., Vitale S.R., Motta G., di Gregorio S., Massimino M., Pennisi M.S., Stella S., Puma A. (2019). Molecular alterations in thyroid cancer: From bench to clinical practice. Genes.

[17] Höppner W., Dralle H., Brabant G. (1998). Duplication of 9 base pairs in the critical cysteine rich domain of the ret proto-oncogene causes multiple endocrine neoplasia type 2A. Hum. Mutat..

[18] Ciampi R., Romei C., Ramone T., Prete A., Tacito A., Cappagli V., Bottici V., Viola D., Torregrossa L., Ugolini C. (2019). Genetic landscape of somatic mutations in a large cohort of sporadic medullary thyroid carcinomas studied by next-generation targeted sequencing. iScience.

[19] Censi S., Cavedon E., Bertazza L., Galuppini F., Watutantrige-Fernando S., de Lazzari P., Nacamulli D., Pennelli G., Fassina A., Iacobone M. (2017). Frequency and significance of ras, tert promoter, and braf mutations in cytologically indeterminate thyroid nodules: A monocentric case series at a tertiary-level endocrinology unit. Front. Endocrinol..

[20] Xing M. (2013). Molecular pathogenesis and mechanisms of thyroid cancer. Nat. Rev. Cancer.

[21] Ranieri G., Marech I., Asabella A.N., di Palo A., Porcelli M., Lavelli V., Rubini G., Ferrari C., Gadaleta C.D. (2017). Tyrosine-kinase inhibitors therapies with mainly anti-angiogenic activity in advanced renal cell carcinoma: Value of PET/CT in response evaluation. Int. J. Mol. Sci..

[22] Oh J.M., Ahn B.-C. (2021). Molecular mechanisms of radioactive iodine refractoriness in differentiated thyroid cancer: Impaired sodium iodide symporter (NIS) expression owing to altered signaling pathway activity and intracellular localization of NIS. Theranostics.

[23] Gild M.L., Tsang V.H.M., Clifton-Bligh R.J., Robinson B.G. (2021). Multikinase inhibitors in thyroid cancer: Timing of targeted therapy. Nat. Rev. Endocrinol..

[24] Brose M.S., Nutting C.M., Jarzab B., Elisei R., Siena S., Bastholt L., de La Fouchardiere C., Pacini F., Paschke R., Shong Y.K. (2014). Sorafenib in radioactive iodine-refractory, locally advanced or metastatic diff erentiated thyroid cancer: A randomised, double-blind, phase 3 Trial. Lancet.

[25] Matsui J., Funahashi Y., Uenaka T., Watanabe T., Tsuruoka A., Asada M. (2008). Multi-kinase inhibitor E7080 suppresses lymph node and lung metastases of human mammary breast tumor MDA-MB-231 via Inhibition of vascular endothelial growth factor-receptor (VEGF-R) 2 and VEGF-R3 kinase. Clin. Cancer Res..

[26] Schlumberger M., Tahara M., Wirth L.J., Robinson B., Brose M.S., Elisei R., Habra M.A., Newbold K., Shah M.H., Hoff A.O. (2015). Lenvatinib versus placebo in radioiodine-refractory thyroid cancer. N. Engl. J. Med..

[27] Locati L.D., Licitra L., Agate L., Ou S.H.I., Boucher A., Jarzab B., Qin S., Kane M.A., Wirth L.J., Chen C. (2014). Treatment of advanced thyroid cancer with axitinib: Phase 2 study with pharmacokinetic/pharmacodynamic and quality-of-life assessments. Cancer.

[28] Carr L.L., Mankoff D.A., Goulart B.H., Eaton K.D., Capell P.T., Kell E.M., Bauman J.E., Martins R.G. (2010). Phase II study of daily sunitinib in FDG-PET-positive, iodine-refractory differentiated thyroid cancer and metastatic medullary carcinoma of the thyroid with functional imaging correlation. Clin. Cancer Res..

[29] Li J., Qin S., Xu J., Guo W., Xiong J., Bai Y., Sun G., Yang Y., Wang L., Xu N. (2013). Apatinib for chemotherapy-refractory advanced metastatic gastric cancer: Results from a randomized, placebo-controlled, parallel-arm, phase ii trial. J. Clin. Oncol..

[30] Lin Y., Wang C., Gao W., Cui R., Liang J. (2017). Overwhelming rapid metabolic and structural response to apatinib in radioiodine refractory differentiated thyroid cancer. Oncotarget.

[31] Adams V.R., Leggas M. (2007). Sunitinib Malate for the treatment of metastatic renal cell carcinoma and gastrointestinal stromal tumors. Clin. Ther..

[32] Schlumberger M., Jarzab B., Cabanillas M.E., Robinson B., Pacini F., Ball D.W., McCaffrey J., Newbold K., Allison R., Martins R.G. (2016). A phase II trial of the multitargeted tyrosine kinase inhibitor lenvatinib (E7080) in advanced medullary thyroid cancer. Clin. Cancer Res..

[33] Haddad R.I., Schlumberger M., Wirth L.J., Sherman E.J., Shah M.H., Robinson B., Dutcus C.E., Teng A., Gianoukakis A.G., Sherman S.I. (2017). Incidence and timing of common adverse events in lenvatinib-treated patients from the select trial and their association with survival outcomes. Endocrine.

[34] Nervo A., Gallo M., Samà M.T., Felicetti F., Alfano M., Migliore E., Marchisio F., Berardelli R., Arvat E., Piovesan A. (2018). Lenvatinib in advanced radioiodine-refractory thyroid cancer: A snapshot of real-life clinical practice. Anticancer. Res..

[35] Fugazzola L., Elisei R., Fuhrer D., Jarzab B., Leboulleux S., Newbold K., Smit J. (2019). 2019 European Thyroid Association guidelines for the treatment and follow-up of advanced radioiodine-refractory thyroid cancer. Eur. Thyroid. J..

[36] Haugen B.R., Alexander E.K., Bible K.C., Doherty G.M., Mandel S.J., Nikiforov Y.E., Pacini F., Randolph G.W., Sawka A.M., Schlumberger M. (2016). 2015 American Thyroid Association management guidelines for adult patients with thyroid nodules and differentiated thyroid cancer: The American Thyroid Association guidelines task force on thyroid nodules and differentiated thyroid cancer. Thyroid.

[37] Basu S., Dandekar M., Joshi A., D’Cruz A. (2015). Defining a rational step-care algorithm for managing thyroid carcinoma patients with elevated thyroglobulin and negative on radioiodine scintigraphy (TENIS): Considerations and challenges towards developing an appropriate roadmap. Eur. J. Nucl. Med. Mol. Imaging.

[38] Silberstein E.B. (2011). The problem of the patient with thyroglobulin elevation but negative iodine scintigraphy: The tenis syndrome. Semin. Nucl. Med..

[39] Schlumberger M., Brose M., Elisei R., Leboulleux S., Luster M., Pitoia F., Pacini F. (2014). Definition and management of radioactive iodine-refractory differentiated thyroid cancer. Lancet Diabetes Endocrinol..

[40] Suzuki C., Kiyota N., Imamura Y., Goto H., Suto H., Chayahara N., Toyoda M., Ito Y., Miya A., Miyauchi A. (2021). exploratory analysis to predict optimal tumor burden for starting lenvatinib in patients with radioiodine-refractory differentiated thyroid cancer. Front. Oncol..

[41] Dohán O., Carrasco N. Advances in Na+/I- Symporter (NIS) Research in the Thyroid and Beyond. Proceedings of the Molecular and Cellular Endocrinology.

[42] Garcia D., Singh V. Nuclear Medicine PET/CT Thyroid Cancer Assessment, Protocols, and Interpretation; [Updated 2021 May 1]. StatPearls Publishing; January 2021. https://www.ncbi.nlm.nih.gov/books/NBK570634/.

[43] Duarte P.S., Marin J.F.G., de Carvalho J.W.D.A., Sapienza M.T., Buchpiguel C.A. (2018). Iodine/FDG “Flip-Flop” Phenomenon Inside a Large Metastatic Thyroid Cancer Lesion Better Characterized on SPECT/CT and PET/CT Studies. Clin. Nucl. Med..

[44] Therasse P., Arbuck S.G., Eisenhauer E.A., Wanders J., Kaplan R.S., Rubinstein L., Verweij J., van Glabbeke M., van Oosterom A.T., Christian M.C. (2000). New guidelines to evaluate the response to treatment in solid tumors. J. Natl. Cancer Inst..

[45] Eisenhauer E.A., Therasse P., Bogaerts J., Schwartz L.H., Sargent D., Ford R., Dancey J., Arbuck S., Gwyther S., Mooney M. (2009). new response evaluation criteria in solid tumours: Revised recist guideline (Version 1.1). Eur. J. Cancer.

[46] Ziai D., Wagner T., el Badaoui A., Hitzel A., Woillard J.B., Melloni B., Monteil J. (2013). Therapy Response Evaluation with FDG-ET/CT in small cell lung cancer: A prognostic and comparison study of the PERCIST and EORTC criteria. Cancer Imaging.

[47] Takeuchi S., Shiga T., Hirata K., Taguchi J., Magota K., Ariga S., Gouda T., Ohhara Y., Homma R., Shimizu Y. (2018). early prediction of lenvatinib treatment efficacy by using 18F-FDG PET/CT in patients with unresectable or advanced thyroid carcinoma that is refractory to radioiodine treatment: A protocol for a non-randomized single-arm multicenter observational study. BMJ Open.

[48] Joo Hyun O., Lodge M.A., Wahl R.L. (2016). Practical percist: A simplified guide to pet response criteria in solid tumors 1.0. Radiology.

[49] Marotta V., Ramundo V., Camera L., del Prete M., Fonti R., Esposito R., Palmieri G., Salvatore M., Vitale M., Colao A. (2013). Sorafenib in advanced iodine-refractory differentiated thyroid cancer: Efficacy, safety and exploratory analysis of role of serum thyroglobulin and FDG-PET. Clin. Endocrinol..

[50] Ahmaddy F., Burgard C., Beyer L., Koehler V., Bartenstein P., Fabritius M.P., Geyer T., Wenter V., Ilhan H., Spitzweg C. (2021). 18F-FDG-PET/CT in patients with advanced, radioiodine refractory thyroid cancer treated with lenvatinib. Cancers.

[51] Vercellino L., Bousquet G., Baillet G., Barré E., Mathieu O., Just P.-A., Desgrandchamps F., Misset J.-L., Hindié E., Moretti J.-L. (2009). 18F-FDG PET/CT imaging for an early assessment of response to sunitinib in metastatic renal carcinoma: Preliminary study. Cancer Biother. Radiopharm..

[52] Caldarella C., Muoio B., Isgrò M.A., Porfiri E., Treglia G., Giovanella L. (2014). The role of Fluorine-18-Fluorodeoxyglucose positron emission tomography in evaluating the response to tyrosine-kinase inhibitors in patients with metastatic primary renal cell carcinoma. Radiol. Oncol..

[53] Wang C., Zhang X., Yang X., Li H., Cui R., Guan W., Li X., Zhu Z., Lin Y. (2018). PET response assessment in apatinib-treated radioactive iodine-refractory thyroid cancer. Endocr. Relat. Cancer.

[54] Manohar P.M., Beesley L.J., Bellile E.L., Worden F.P., Avram A.M. (2018). Prognostic value of FDG-PET/CT metabolic parameters in metastatic radioiodine-refractory differentiated thyroid cancer. Clin. Nucl. Med..

[55] Valerio L., Guidoccio F., Giani C., Tardelli E., Puccini G., Puleo L., Minaldi E., Boni G., Elisei R., Volterrani D. (2021). [18F]-FDG-PET/CT correlates with the response of radiorefractory thyroid cancer to lenvatinib and patient survival. J. Clin. Endocrinol. Metab..

[56] Ahn S., Kim T.H., Kim S.W., Ki C.S., Jang H.W., Kim J.S., Kim J.H., Choe J.H., Shin J.H., Hahn S.Y. (2017). Comprehensive screening for PD-L1 expression in thyroid cancer. Endocr. Relat. Cancer.

[57] Na K.J., Choi H. (2018). Immune Landscape of papillary thyroid cancer and immunotherapeutic implications. Endocr. Relat. Cancer.

[58] Basu S., Joshi A. (2016). 68Ga Dotatate Pet/Ct in Differentiated Thyroid Carcinoma with Fibular Metastasis and Mixed Response to Sorafenib. Clin. Nucl. Med..

